# A rare co-occurrence of duchenne muscular dystrophy, congenital adrenal hypoplasia and glycerol kinase deficiency due to Xp21 contiguous gene deletion syndrome: case report

**DOI:** 10.1186/s12902-021-00876-6

**Published:** 2021-10-24

**Authors:** Asanka Rathnasiri, Udara Senarathne, Visvalingam Arunath, Thabitha Hoole, Ishara Kumarasiri, Oshanie Muthukumarana, Eresha Jasinge, Sachith Mettananda

**Affiliations:** 1 Colombo North Teaching Hospital, Ragama, Sri Lanka; 2 Department of Biochemistry, Faculty of Medical Sciences, University of Sri Jayewardenepura, Nugegoda, Sri Lanka; 3 Lady Ridgeway Children’s Hospital, Colombo 08, Colombo, Sri Lanka; 4 Department of Paediatrics, Faculty of Medicine, University of Kelaniya, Thalagolla Raod, Ragama, Sri Lanka

**Keywords:** Contiguous gene deletion syndrome, Congenital adrenal hypoplasia, Duchenne muscular dystrophy, Glycerol kinase deficiency

## Abstract

**Background:**

Contiguous gene deletion syndromes are rare genomic disorders caused by deletion of large segments of DNA resulting in co-occurrence of apparently unrelated multiple clinical phenotypes. We report a boy with contiguous gene deletion involving Xp21 genomic location.

**Case presentation:**

A Sri Lankan boy with developmental delay and failure to thrive first presented at three years of age with hypovolaemia, hyperpigmentation and drowsiness. Investigations done at that time revealed hypoglycaemia, hyponatraemia, hyperkalaemia, low cortisol, low aldosterone, high ACTH and low 17-hydroxyprogesterone. He was diagnosed to have primary adrenal insufficiency.

During follow-up at five years, he was noted to have progressive difficulty in walking, waddling gait, hypotonia, calf hypertrophy and positive Gower’s sign. His creatine kinase was very high, and the electromyogram showed myopathy. Genetic analysis revealed hemizygous deletion involving the final 35 exons of the dystrophin gene confirming the diagnosis of Duchenne muscular dystrophy.

Further investigations revealed pseudohypertriglyceridemia, large glycerol peak on urine organic acid analysis and hemizygous deletion of the glycerol kinase gene confirming glycerol kinase deficiency. Based on the presence of Duchenne muscular dystrophy, glycerol kinase deficiency and probable congenital adrenal hypoplasia along with genetic confirmation of deletions involving dystrophin and glycerol kinase genes, the diagnosis of Xp21 contiguous gene deletion syndrome was made.

**Conclusions:**

We report a child with contiguous gene deletion syndrome who was initially diagnosed as having isolated primary adrenal insufficiency probably due to congenital adrenal hypoplasia. Later he was confirmed to have Duchenne muscular dystrophy and glycerol kinase deficiency, as well. This case report highlights the importance of pre-emptive evaluation and identification of genetic defects when patients present with seemingly unrelated diseases that could aid in accurate diagnoses of contiguous gene deletion syndromes.

## Background

Contiguous gene deletion syndromes are rare genomic disorders caused by deletion of large segments of DNA containing several contiguous genes [1, 2]. They result in the co-occurrence of apparently unrelated multiple clinical phenotypes due to defects in proteins expressed by genes which are located in close proximity to each other [2]. Here, we report a boy with three distinct clinical entities - Duchenne muscular dystrophy, congenital adrenal hypoplasia and glycerol kinase deficiency - due to contiguous gene deletion involving Xp21 genomic location.

## Case presentation

A Sri Lankan boy was first admitted to the paediatric ward at the age of 3 years with complaints of difficulty in feeding, failure to thrive and global developmental delay. He was born to non-consanguineous parents at term with a birth weight of 2.5 kg and did not have significant neonatal complications. On examination, he was drowsy, had hyperpigmentation involving perioral, buccal and palmar areas and showed evidence of hypovolaemia with tachycardia and low volume pulse.

Investigations performed at that time revealed; serum sodium 120mmol/L, potassium 7.1mmol/L, glucose 30 mg/dL, cortisol 4nmol/L (normal 120-626), ACTH 343pg/mL (normal 7-41), aldosterone 0.97ng/dL (normal 1.76-23.2), plasma renin concentration 254µIU/mL (normal 2.8-32.9), plasma renin activity 21ng/mL/hr (normal 1.9-5.2), cholesterol 153 mg/dL (normal 125-170), 17-hydroxyprogesterone 1.1ng/dL (normal 3-90) and dehydroepiandrosterone sulphate 0.08µmol/L (normal 0.7-5.7). Serum levels of thyroid stimulating hormone and abdominal ultrasonography were normal. Based on the biochemical features of hyponatraemia, hyperkalaemia, hypoglycaemia, low cortisol, low aldosterone, high ACTH in the presence of low 17-hydroxyprogesterone and dehydroepiandrosterone sulphate levels, he was diagnosed to have primary adrenal insufficiency due to a defect in the initial steps of adrenal hormone biosynthesis or congenital adrenal hypoplasia. He was commenced on oral hydrocortisone and fludrocortisone.

**Fig. 1:**
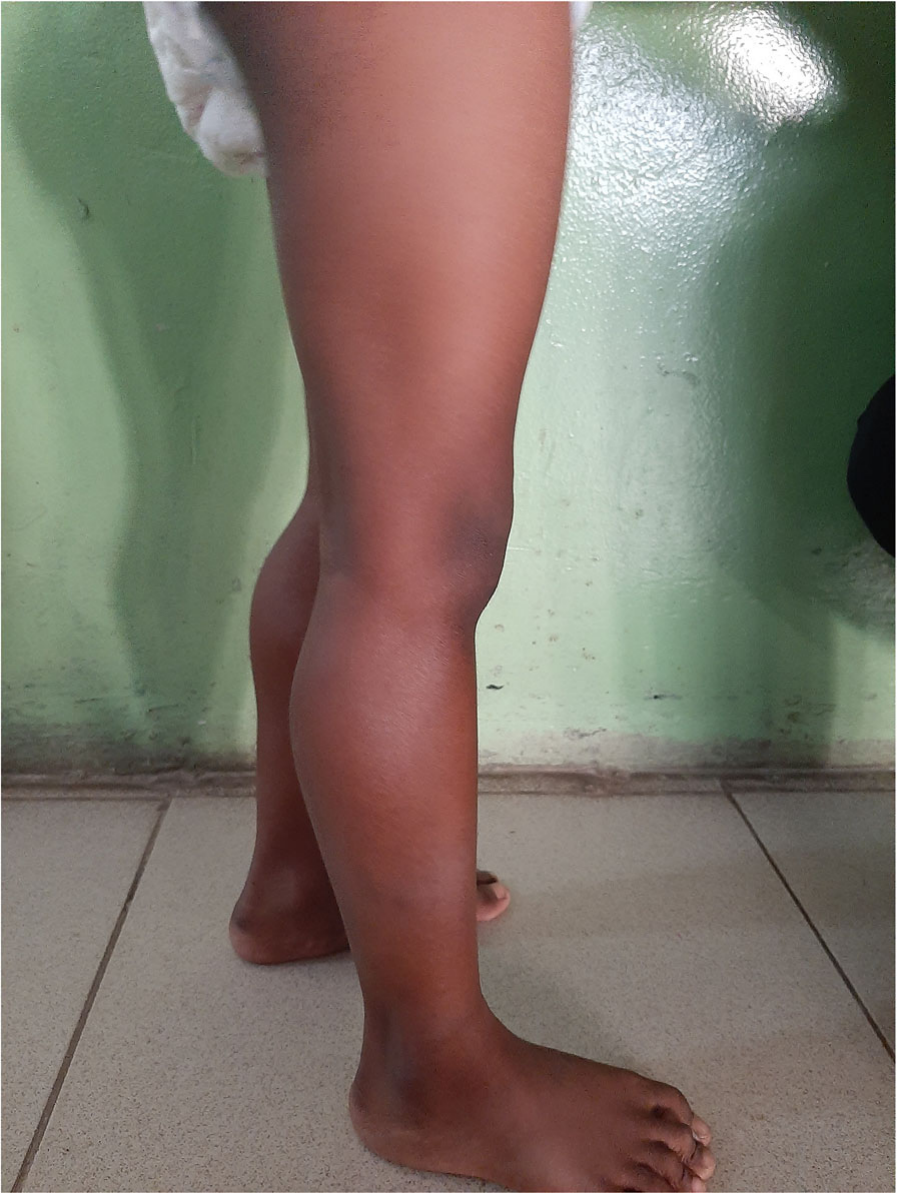
Photograph of the patient showing pseudohypertrophy of the calf

At the age of 5 years, he was admitted again with a history of difficulty in walking and getting up from the seated position, which was gradually worsening. Physical examination at this point revealed bilateral calf hypertrophy, waddling gait, positive Gower’s sign, hypotonia of both lower limbs, muscle power of grade 4 and normal tendon reflexes suggestive of a classical proximal myopathy (Fig. 1). His creatine kinase was 12,395U/L (normal 38-174), and electromyography showed evidence of myopathy. His electrocardiography and echocardiogram were normal. Genetic mutation analysis using multiplex ligation-dependent probe amplification method revealed hemizygous deletion encompassing final 35 exons (exon 45 to 79) of the dystrophin gene confirming the diagnosis of Duchenne muscular dystrophy.

**Fig. 2:**
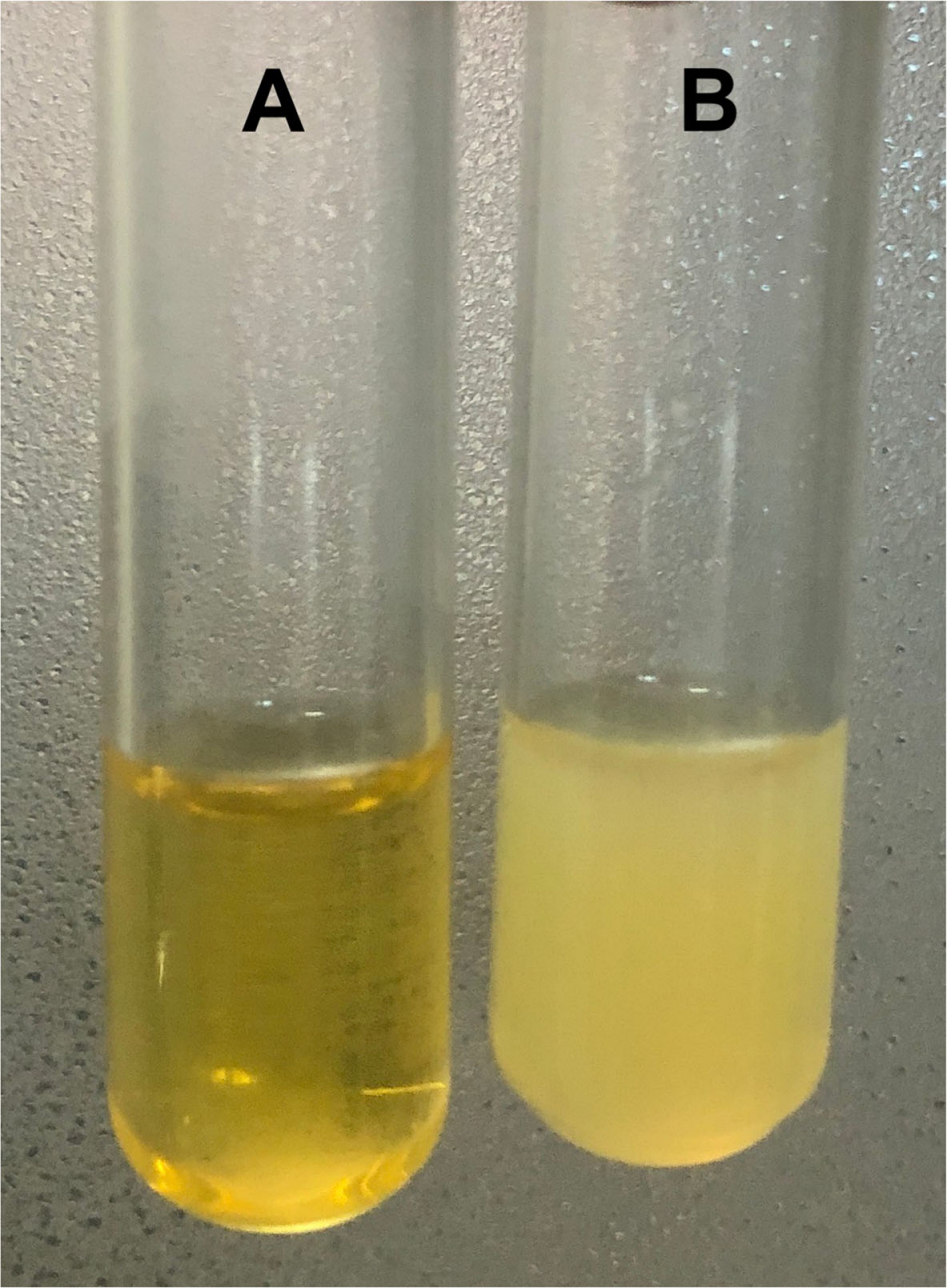
Photographs of serum samples of this patient (triglyceride 4.0 mmol/L) with clear appearance (**A**) and that of a patient with true hypertriglyceridemia (triglyceride 2.1 mmol/L) with turbid appearance (**B**)

**Fig. 3:**
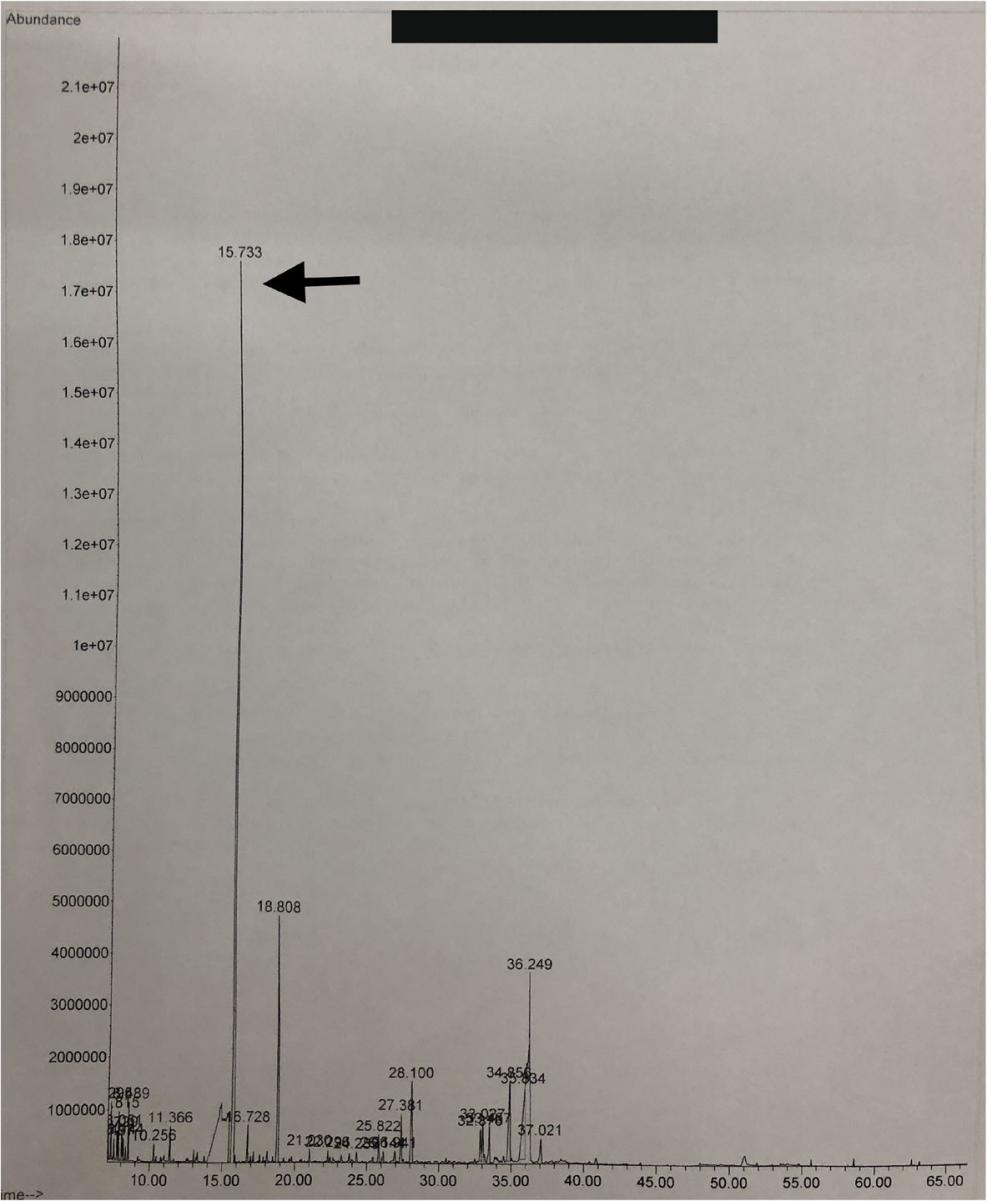
Urinary organic acid analysis showing glycerol peak (arrow)

Due to the co-occurrence Duchenne muscular dystrophy and primary adrenal insufficiency probably due to congenital adrenal hypoplasia, Xp21 contiguous gene deletion syndrome was suspected. Further investigations revealed hypertriglyceridemia (4.0 mmol/L), which was associated with non-turbid serum leading to the suspicion of pseudohypertriglyceridemia due to the presence of free glycerol in serum (Fig. 2). This was confirmed by the detection of a large glycerol peak on the urinary organic acid analysis using gas chromatography-mass spectrometry (Fig. 3). Genetic mutation testing by next generation sequencing based copy number variation analysis confirmed glycerol kinase deficiency due to hemizygous deletion encompassing the entire glycerol kinase gene (exons 1 to 21). Genetic testing for congenital adrenal hypoplasia was not done due to unavailability.

Based on the presence of Duchenne muscular dystrophy, glycerol kinase deficiency and probable congenital adrenal hypoplasia along with genetic confirmation of deletions involving dystrophin and glycerol kinase genes, the diagnosis of Xp21 contiguous gene deletion syndrome was made. He was started on oral prednisolone 0.75 mg/kg daily as a treatment for Duchenne muscular dystrophy; oral hydrocortisone was discontinued, but fludrocortisone was continued. Family screening revealed intellectual disability and elevated creatine kinase level [1108 U/L (normal 26-140)] in his mother suggesting heterozygous carrier state. His only sibling, who was a 13-year old boy was clinically normal and none of the member in the extended family were affected.

## Discussion and conclusions

The Xp21 contiguous gene deletion syndrome is a rare disorder characterised by co-occurrence of congenital adrenal hypoplasia, Duchenne muscular dystrophy, chronic granulomatous disease, retinitis pigmentosa, ornithine transcarbamylase deficiency, glycerol kinase deficiency and mental retardation [3–6]. Presence of two or more features suggests the diagnosis [5]. Our patient had congenital adrenal hypoplasia, Duchenne muscular dystrophy and glycerol kinase deficiency thus confirming the diagnosis of Xp21 contiguous gene deletion syndrome.

The index patient presented initially with features of primary adrenal insufficiency. The diagnosis of congenital adrenal hypoplasia was made when he was genetically confirmed to have Duchenne muscular dystrophy and glycerol kinase deficiency at 5 years. Amongst multiple aetiologies of congenital adrenal hypoplasia, the commonest mutation affects the *NR0B1/DAX1* gene which is located in Xp21.2 genomic region [7]. Although mutation analysis was not performed for congenital adrenal hypoplasia in this child, the most likely genetic defect is a large deletion involving the *NR0B1/DAX1* gene along with dystrophin and glycerol kinase genes, which are located in close proximity to one another.

Glycerol kinase deficiency in the index patient was diagnosed following pre-emptive investigation performed due to a tentative diagnosis of Xp21 contiguous gene deletion syndrome. Glycerol kinase (IUB: 2.7.1.30) is the enzyme responsible for phosphorylation of glycerol from triglyceride breakdown for further metabolism. The absence of this enzyme activity leads to the accumulation of glycerol in circulation, causing glycerolaemia and glyceroluria [8, 9]. The glycerolaemia is usually detected as pseudohypertriglyceridemia due to overestimation of serum triglyceride levels as a result of analytical interference by free glycerol on the assay method as observed in this patient [10]. The measurement of fasting serum triglyceride level along with visual observation of serum can be used to screen for pseudohypertriglyceridemia due to glycerolaemia. Therefore, we suggest performing this simple test in children with primary adrenal insufficiency without obvious etiological diagnoses to rule out concurrent glycerol kinase deficiency.

Hypoglycaemia is a feature in both congenital adrenal hypoplasia and glycerol kinase deficiency. In congenital adrenal hypoplasia, hypoglycaemia is due to the deficiency of counterregulatory hormone cortisol. In glycerol kinase deficiency, the conversion of glycerol to glycerol-3-phosphate is impaired thus limiting substrate for gluconeogenesis. Thus, in Xp21 contiguous gene deletion, hypoglycaemia is a combine effect of congenital adrenal hypoplasia and glycerol kinase deficiency.

In conclusion, we report a child with contiguous gene deletion syndrome who was initially diagnosed as having isolated primary adrenal insufficiency probably due to congenital adrenal hypoplasia. This case report highlights the importance of identifying the exact genetic defects when patients present with seemingly unrelated genetic diseases which could lead to accurate diagnoses of contiguous gene deletion syndromes.

## Data Availability

Not applicable.

## Abbreviations

DNA: Deoxyribonucleic acid
